# Innovative use of wild Egyptian artichoke extract to control fowl cholera *in vitro*

**DOI:** 10.14202/vetworld.2025.341-347

**Published:** 2025-02-13

**Authors:** Ali Wahdan, Mahmoud Fahmi Elsebai, Mahmoud M. Elhaig, Ibrahim M. El-Sabagh, Mohamed S. Ahmed, Mahmoud Mohamed, Ehab M. Abd-Allah

**Affiliations:** 1 Department of Bacteriology, Immunology, and Mycology, Faculty of Veterinary Medicine, Suez Canal University, Ismailia, 41522, Egypt; 2 Department of Pharmacognosy, Faculty of Pharmacy, Mansoura University, Mansoura, Egypt; 3 Department of Animal Medicine (Infectious Diseases), Faculty of Veterinary Medicine, Suez Canal University, Ismailia, 41522, Egypt; 4 Central Biotechnology Laboratory, Collage of Veterinary Medicine, King Faisal University, Al-Ahsa, 31982, Saudi Arabia; 5 Department of Virology, Faculty of Veterinary Medicine, Cairo University, 12211, Giza, Egypt; 6 Department of Clinical Sciences, College of Veterinary Medicine, King Faisal University, Al-Ahsa 31982, Saudi Arabia; 7 Department of Poultry Diseases, Faculty of Veterinary Medicine, South Valley University, Qena, Egypt; 8 Department of Avian and Rabbit Medicine, Faculty of Veterinary Medicine, 44511 Zagazig University, Egypt; 9 Veterinary Hospital, Faculty of Veterinary Medicine, Zagazig University, Zagazig 44511, Egypt

**Keywords:** fowl cholera, gene expression, multidrug resistance, *Pasteurella multocida*, virulence genes, wild Egyptian artichoke

## Abstract

**Background and Aim:**

Fowl cholera, caused by multidrug-resistant (MDR) *Pasteurella multocida* type A, poses a significant threat to chicken production globally. This study investigates the potential of ethanolic extracts from Wild Egyptian Artichoke (WEA) (*Cynara cardunculus* L. var. *sylvestris*) to modulate virulence-associated genes and provide an alternative control strategy.

**Materials and Methods:**

A total of 160 tissue samples from diseased chickens were collected and analyzed. Phenotypic, biochemical (via Vitek 2 Compact), and molecular methods were used to identify *P. multocida*. Polymerase chain reaction (PCR) confirmed the presence of key adhesion and colonization genes (*omp87*, *ptfA*, *pfhA*) in MDR isolates. The antimicrobial efficacy of WEA ethanolic extract was assessed using disk diffusion, minimum inhibitory concentration (MIC), and minimum bactericidal concentration (MBC) assays. Gene expression changes were evaluated using quantitative reverse-transcription PCR after treatment with sub-inhibitory extract concentrations.

**Results:**

Eleven isolates (6.9% prevalence) of *P. multocida* type A were confirmed, with four showing resistance to over five antimicrobial classes. The ethanolic WEA extract demonstrated significant antibacterial activity, with inhibition zones of up to 25 mm, MIC values ranging from 4 to 16 µg/mL, and MBC values between 8 and 32 µg/mL. Gene expression analysis revealed up to threefold downregulation in *omp87* (0.28-fold), *pfhA* (0.25-fold), and *ptfA* (0.12-fold) after treatment.

**Conclusion:**

The WEA ethanolic extract effectively downregulates critical virulence genes in MDR *P. multocida*, highlighting its potential as a novel natural agent for controlling fowl cholera in chicken. This study emphasizes the importance of exploring plant-based antimicrobials to combat resistance and improve animal health.

## INTRODUCTION

*Pasteurella multocida* is a Gram-negative bacterium that poses a threat to the global chicken industry and inflicts enormous economic losses because of the haphazard application of antimicrobial drugs and inadequate vaccination programs [1]. *P. multocida* causes fowl cholera in avians, hemorrhagic septicemia in bovines, rhinitis in swine, and snuffles in rabbits [1]. Fowl cholera is caused by *P. multocida* type A, which normally inhabits the upper respiratory tract. It is not clear how modifications can be harmful and induce illness [2]. The primary ingredient of *P. multocida* type A capsules is hyaluronic acid, which enhances growth by supporting the formation of extracellular matrix [3].

Most pathogens require adherence to the host’s epithelial tissues to initiate infection and illness. This stage can be made possible by the genes that inhabit bacteria, which allow them to colonize and release poisons and enzymes that give them aggressiveness and toxicity [4]. Reverse transcription polymerase chain reaction (RT-PCR) was used to assess the expression levels of adhesion and invasion virulence genes [5]. Predisposing conditions are typically necessary for *P. multocida* to arise as a primary disease, and these factors can increase disease severity. The virulence of *P. multocida* encoding adhesions (*ptfA*, *pfhA*, and *omp87*) differs between strains and host species [6]. Almost all Gram-negative bacteria develop resistance to plasmids containing various resistance genes. The existence of multidrug-resistant (MDR) pathogens highlights the urgent need for appropriate medicines, including natural plant extracts with antibacterial properties [7]. The antiviral activity of Wild Egyptian Artichoke (WEA) against hepatitis C virus (HCV) was reported for the 1^st^ time [8]. Another study by Elsebai *et al*. [9] presented comprehensive details on the chemistry of WEA metabolites and assessed their capacity to inhibit HCV *in vitro*. It was discovered that six compounds, particularly cynaropicrin and grosheimol, were effective in preventing HCV entry into cells. Moreover, chloropicrin has antihyperlipidemic, antimicrobial, and anti-inflammatory properties. The antibacterial activity of cynaropicrin was achieved by permanently inhibiting MurA enzyme, which is crucial for the bacterial cytoplasmic manufacture of precursors for peptidoglycan [10].

Little research has focused on the role of herbs against *P. multocida* strains [11]. However, no studies have discussed the role of WEA in controlling the expression of the *P. multocida* genes. Thus, this study aimed to retard the pathogenic step and decrease or prevent drug resistance by *P. multocida* strains using WEA extract.

## MATERIALS AND METHODS

### Ethical approval

Handling, preparation, and disposal of the materials were approved by the Scientific Research Ethics Committee of the Faculty of Veterinary Medicine, Suez Canal University (SCU2024003).

### Study period and location

The study was conducted from January 2019 to October 2020. The samples were collected from different private farms in an eastern area of Dakahlia, Egypt and processed at the Bacteriology Department, Faculty of Veterinary Medicine, Suez Canal University

### Sampling

A total of 160 diseased chickens aged over 2 months who were not vaccinated against fowl cholera were used. The chickens comprised of 80 diseased layers and 80 diseased broilers from different private farms in an eastern area of Dakahlia, Egypt. The chickens were reared under an intensive system, fed commercial rations containing 18% protein, and examined for *P. multocida* infection. The examined chickens had respiratory signs and weakness. Different organs from each chicken were combined and displayed as a single tissue sample and transported in peptone water for bacteriological examination.

### Isolation and Vitek 2 compact (bioMérieux, France) confirmation

Samples from different organs were cultivated on sheep blood agar (Oxoid, UK) and MacConky agar (Oxoid) and incubated at 37°C for 18–24 h [12]. The recovered colonies were inspected morphologically and biochemically using a Vitek 2 compact system (bioMérieux). In summary, colonies of identified Gram-negative bacilli were suspended in 3 ml of Vitek 2 saline solution using an automated Vitek 2 Densichek (bioMérieux) until the turbidity reached 0.52–0.62. Gram-negative cards were placed on cassettes along with appropriate samples [13]. Each isolate was kept at −80°C in Tryptic soya broth for further analysis.

### Molecular confirmation and typing of *P. multocida*

DNA from isolates verified using the Vitek 2 compact (bioMérieux) was extracted using a QIAprep^®^Spin kit (Cat. 27104, Qiagen, Germany). Conventional PCR was performed to amplify 460 bp using the species-specific *Kmt1* gene of *P. multocida* (KMT1T7: 5′-ATCCGCTATTTACCCAGTGG-3′, KMT1SP6: 5′ GCTGTAAACGAACTCGCCAC-3′) [14]. For the capsular typing of *P. multocida* isolates, multiplex PCR targeting capsular genes using specific primers was performed under thermal cycling conditions as previously described by Townsend *et al*. [14] and Malik *et al*. [15]. *P. multocida* *sub multocida* ATCC, 12945^™^ (ATCC, USA) was used as a positive control, and nuclease-free water was used as a negative control, one cycle at 95°C for 5 min, followed by 30 cycles at 95°C for 30 s, 55°C for 30 s, 72°C for 30 s, and a final cycle at 72°C for 5 min. PCR products were separated by gel electrophoresis and visualized by a gel documentation system (Biospectrum UVP, UK).

### Antimicrobial-resistant profile

The antimicrobial resistance pattern of the identified *P. multocida* type A was tested against different widely commercially used antibacterial groups according to Clinical and Laboratory Standards Institute (CLSI) [16] using the disk diffusion method. The antibacterial agents (Oxoid) tested were amoxicillin + clavulanic acid 20/10 µg (AMC), doxycycline 30 µg (Do), colistin 10 µg (CT), cefotaxime 30 µg (CTX), ciprofloxacin 5 µg (CIP), tobramycin 10 µg (TOB), gentamicin 10 µg (CN), erythromycin 15 µg (E), nitrofurantoin 300 µg (F), and chloramphenicol 30 µg (C). The diameter of the inhibition zone matched the zone diameter scheduled by the CLSI [16]. *P. multocida* strains that expressed resistance to different antimicrobials were considered in further examination.

### Molecular detection of encoding adhesion genes

The existence of adhesion pathogenic genes (*omp87* [438 bp], *ptfA* [488 bp], and *pfhA* [286 bp]) was investigated by PCR using primers and amplification conditions in four isolates (P.3, P.6, P.7, and P.10) that showed broad resistance to antibiotics, as described by Karthik *et al*. [17].

### Preparation of ethanolic WEA leaf extracts

The process of WEA extraction from freshly washed leaves required adding ethyl alcohol (75%) at a 1:10 w/v ratio, grinding the material fully using an electric mixer, and filtering both extracts using Whatman No. 2 filter paper. The portion of ethanolic extract evaporated after being subjected to a rotating evaporator at 35°C was determined as previously described by Nigussie *et al*. [18]. All metabolites of WEA were previously reported by Elsebai *et al*. [8].

### WEA ethanolic extract activity against extensive resistant *P. multocida* strains

The efficacy of the ethanolic WEA extract was evaluated against four highly pathogenic and widely resistant isolates using the agar diffusion method. Two sterile filter papers with a diameter of 6 mm were used: One was soaked in ethanolic extract at different concentrations (256 µg/mL, 128 µg/mL, 64 µg/mL, 32 µg/mL, 16 µg/mL, 8 µg/mL, 4 µg/mL, and 2 µg/mL) and the other was soaked in distilled water as negative control. The concentrations were adjusted as described by Elsebai *et al*. [8]. The broth microdilution method was used to calculate the minimum inhibitory concentration (MIC) and minimum bactericidal concentration (MBC) readings [19].

### Measurement of the messenger RNA (mRNA) expression of *P. multocida* encoding genes

The sub-inhibitory concentration of the ethanolic WEA extract was incubated with resistant virulent *P. multocida* for >12 h [20], and total RNA was extracted using a PureLink^™^ RNA kit (Cat.12183018A, Invitrogen, USA). The expression of mRNA was identified using one-step qRT-PCR in a 25-µL reaction volume and performed in duplicate. The expressed mRNA levels of the concerned genes were standardized according to intrinsic expression of the housekeeping gene. The levels of genes encoding the treated strains were expressed as fold changes [11].

### Statistical analysis

The data were analyzed using GraphPad Prism 9 (GraphPad Software Inc., USA). All experiments were performed in triplicate, and the results are expressed as mean ± standard deviation. The differences in gene expression levels (*omp87, ptfA*, and *pfhA*) before and after treatment with the WEA ethanolic extract were analyzed using a paired Student’s t-test. A one-way analysis of variance followed by Tukey’s post hoc test was used to compare the inhibition zones, MIC, and MBC values across different isolates. Statistical significance was set at p < 0.05.

## RESULTS

Eleven *P. multocida* strains were recovered from 160 diseased chicken, representing a percentage of 6.9%. The highest isolation rate of *P. multocida* was observed in 8.75% (7/80) of diseased layers, followed by diseased broilers (5%, 4/80). On MacConkey medium (Oxoid), *P. multocida* did not grow, and on blood medium (Oxoid), it produced small, smooth colonies lacking hemolytic activity. Gram-negative coccobacilli were observed under a ordinary microscope (Olympus corporation, Japan) at 100x. All 11 *Pasteurella* isolates were biochemically confirmed to be *P. multocida* using the Vitek 2 compact (bioMérieux) with a probability of 95%–99% (excellent identification).

**Figure 1:**
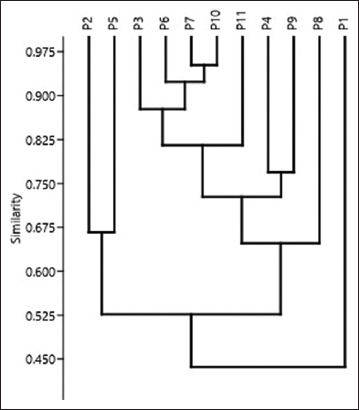
Antimicrobial resistance clustering of isolated *Pasteurella multocida* against different used antimicrobial agents by multivariate analysis.

All recovered field *P. multocida* strains showed high resistance to CT, Do, CTX, erythromycin, chloramphenicol, amoxicillin +clavulanic acid, and gentamicin at 90.9%, 81.8%, 81.8%, 81.8%, 72.7%, 63.6%, and 63.6%, respectively. Resistance to CIP, vancomycin, nitrofurantoin, and TOB was 54.5%, 45.45%, 36.36%, and 36.36%, respectively. The multivariate analysis in Figure 1 showed that four isolates (numbered P3, P6, P7, and P10) were resistant to more than five antimicrobials. These isolates were selected to determine their sensitivity to the ethanolic WEA extract.

PCR targeting the *kmt1* gene (460bp) was used to confirm the identity of *P. multocida* among the isolates screened by Vitek 2. Using hyaD-hyaC PCR (1044bp), all isolates were classified as *P. multocida* type A. All four extensive field-resistance *P. multocida* type A strains were found to carry the attachment and colonization virulent genes *ptfA*, *pfhA*, and *omp87* at 200, 488, and 830 bp, respectively.

The inhibition zone diameter, MIC, and MBC of the ethanolic WEA extract were measured to assess its potential actions against four MDR *P. multocida* isolates harboring the virulent genes for attachment and colonization, as indicated in Table 1. The ethanolic extract of WEA showed a high inhibition zone at 25 mm, the MIC reached 4 µg/mL, and the MBC ranged from 8 to 32 µg/mL.

**Table 1:** Ethanolic WEA extract activity against virulent MDR *Pasteurella multocida* isolates.

Isolate number	Inhibition diameter (mm)	Ethanolic WEA extract concentrations (µg⁄mL)								MIC (µg⁄mL)	MBC (µg⁄mL)

		256	128	64	32	16	8	4	2		
*p. 3*	25	-	-	-	-	-	-	+	+	4.0	8.0
*p. 6*	19	-	-	-	-	+	+	+	+	16.0	32.0
*p. 7*	17	-	-	-	-	+	+	+	+	16.0	32.0
*p. 10*	23	-	-	-	-	-	+	+	+	8.0	16.0

WEA=Wild Egyptian Artichoke, MDR=Multidrug resistance, MIC: Minimum inhibitory concentration, MBC: Minimum bactericidal concentration

**Figure 2:**
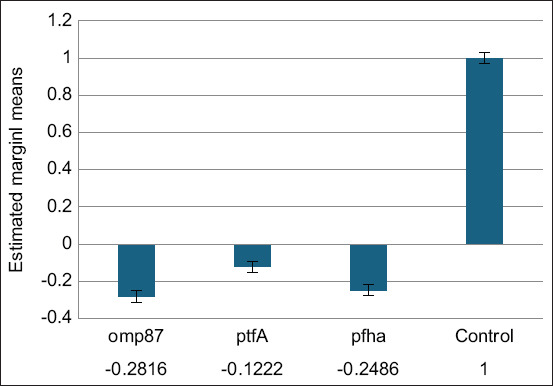
*Omp87*, *ptfA*, and *pfhA* gene expression levels following treatment with a subinhibitory dose of ethanolic wild Egyptian artichoke extract.

The expression levels of the chosen virulence genes (*omp87*, *ptfA*, and *pfhA*) of *P. multocida* were assessed by PCR following treatment with a sub-inhibitory quantity of WEA extract to understand the effect of the ethanolic WEA extract on these genes. The three virulence genes that were studied had varying degrees of downregulation following exposure, as shown in Figure 2. The genes of interest showed up to three-fold downregulation, 0.2816-fold downregulation for omp87, 0.2486-fold downregulation for *pfh*A, and 0.1222-fold downregulation for *ptf*A, compared with the untreated control strains.

## DISCUSSION

This study aimed to determine the effect of WEA extract on *P. multocida* strains isolated from chicken flocks in Egypt and to determine the expression of the *omp87*, *ptfA*, and *pfhA* genes involved in the modification of the pathogenicity of *P. multocida*.

In this study, the prevalence of *P. multocida* was 6.9% using bacteriological methods, which is comparable to the 7.6% prevalence reported using the same assay in Upper Egypt [21] and higher than 1.2% reported from chicken farms in Nigeria [22]. On the other hand, the isolation rate was lower than 13.04% and 59.72% reported in Bangladesh [23, 24]. The variations in the prevalence of *P. multocida* may be related to the samples taken from individual instances or several farms, the hygienic practices within each farm, and the unlimited application of many antimicrobials during the chicken raising process [1].

Vitek 2 compact and PCR confirmed the 11 *Pasteurella* isolates as *P. multocida*. These isolates had a consistent amplicon size of 1044 bp, indicating that they were all members of capsular group A. This study lends credence to previous reports by Christensen and Bisgaard [1] and Shehata and Hafez [25] that fowl cholera is mainly caused by *P. multocida*, belonging to capsular group A. On the other hand, previous studies by Saha *et al*. [26] and Saha *et al*. [27] have reported that other capsular groups are the causes of fowl cholera in avian species.

Emerging *P. multocida* strains exhibit high resistance to CT, doxycycline, CTX, erythromycin, chloramphenicol, AMC acid, and gentamicin. These results are in close agreement with Mahrous *et al*. [2], who reported that all examined *P. multocida* collected from rabbits were MDR, and Yoshimura *et al*. [28] reported that cefotaxime, CIP, and chloramphenicol were the best antimicrobials used to treat pasteurellosis. The primary cause of MDR strains in Egypt is improper utilization of antibiotics without following a controlled regimen, in addition to resistance genes expressed on plasmids that spread from animals to humans and between various species of bacteria [29].

Four MDR *P. multocida* strains were examined and revealed *omp87, ptfA*, and *pfhA*. Because of their ability to connect to the receptors on chicken epithelial cells, these genes are essential for the development and pathophysiology of illness. Prajapati *et al*. [30] demonstrated that the *ptfA*, *pfhA*, *omp87*, *nanB*, and *toxA* genes are present in all *P. multocida* strains isolated from different animal species.

The results shown in Figure 2 are in close agreement with those of Scavo *et al*. [31], who explained that the most effective solvent against certain Gram-positive and Gram-negative bacteria was the artichoke ethanolic extract, followed by the methanolic and water extracts. The ethanolic extract had a higher concentration of cynaropicrin than the water and methanolic extracts. As a therapeutic food plant, artichokes exhibit various pharmacological effects. For example, they have antibacterial properties because of the irreversible inhibition of MurA enzyme, which is crucial for the manufacture of peptidoglycan in bacterial cells [8]. Compared with the water extract, the ethanolic extract contains only the flavonoid apigenin, which increases its efficacy against infections resistant to quinolones [32, 33]. Compared with the other extracts, luteolin 7-O-glucoside and caffeoylquinic acids significantly affected the Gram-positive bacteria in significant amounts in the ethanolic extract [34]. Gram-negative bacteria are generally more tolerant than Gram-positive microorganisms because of their permeability in cell membranes and the existence of periplasmic zones harboring digestive enzymes [35].

The expression of the tested virulence genes after incubation with WEA showed up to 3-fold downregulation: 0.2816-fold for *omp87*, gene 0.2486-fold for *pfhA*, and gene and 0.1222-fold for *ptfA* gene compared with the untreated control strains. Our results agree with those of Abd El-Hamid *et al*. [11], who examined the effect of marjoram on *P. multocida* encoding the *nanB*, *ptfA*, *pfhA*, *toxA*, and *omp87* genes by qRT-PCR. The findings revealed that *ptfA*, *pfhA*, *toxA*, and *omp87* genes were downregulated from 0.214 to 0.4258-fold, while *nanB* was not significantly downregulated. One plausible theory for the mechanism by which herbal extracts mediate their antimicrobial activities is that they interfere with membrane activities and interact with the constituents of membrane proteins, resulting in structural and functional disruption [36]. As previously reported by Kha and Le [37], another theory linked the antibacterial properties of extracts to the destruction of bacterial extracellular enzymes. Given that a combination of bioactive compounds can influence many targets of the *P. multocida* bacterium, it makes sense to expect that a combination of herbal components and antimicrobials would have more bioactivity than a single agent [38].

## CONCLUSION

This study highlights the potential of WEA ethanolic extract as an innovative natural alternative for controlling MDR *P. multocida* type A, a significant pathogen responsible for fowl cholera in chicken. The extract exhibited notable antibacterial activity, with inhibition zones of up to 25 mm, MIC values as low as 4 µg/mL, and MBC values ranging from 8 to 32 µg/mL. Gene expression analysis revealed significant downregulation of critical virulence genes (*omp87, pfhA, ptfA*), with reductions of up to threefold compared to untreated strains. These findings suggest that the WEA extract can modulate virulence pathways, thereby reducing pathogenicity.

The study provides a novel approach to tackling antimicrobial resistance in *P. multocida* using a plant-based extract, with quantitative insights into gene expression changes offering robust evidence of the extract’s mechanism of action. However, the study was conducted in vitro, which may not fully replicate the in vivo environment of chicken flocks, and the sample size of resistant *P. multocida* isolates (n = 4) was limited, potentially affecting the generalizability of the findings. While the study identifies the bioactivity of WEA extract, individual bioactive compounds were not tested separately for their specific contributions.

*In vivo* trials are essential to validate the efficacy and safety of WEA extract in chickens and determine optimal dosages. Isolation and testing of individual bioactive compounds within the WEA extract could help identify specific molecules responsible for antimicrobial and anti-virulence effects. Further research should explore the potential synergistic effects of combining WEA extract with conventional antibiotics to enhance efficacy against MDR pathogens. Expanding the study to other bacterial pathogens of veterinary importance can broaden the application of WEA as a natural antimicrobial agent.

In conclusion, WEA ethanolic extract holds promise as a cost-effective and eco-friendly solution for managing fowl cholera and other MDR infections, paving the way for its incorporation into veterinary practice and antimicrobial stewardship programs.

## AUTHORS’ CONTRIBUTIONS

AW, MFE, and MME: Conceptualized and designed the study. IME, MSA, and MM: Collected the samples and analyzed the data. AW, MME, and EMA: Performed detection of *P. multocida* and antimicrobial sensitivity analyses. AW and MME: Drafted the manuscript. All authors have read and approved the final manuscript.
